# Platypnea-orthodeoxia associated with a fenestrated atrial septal aneurysm: Case Report

**DOI:** 10.1186/1476-7120-3-28

**Published:** 2005-09-13

**Authors:** William J van Gaal, Majo Joseph, Elizabeth Jones, George Matalanis, Mark Horrigan

**Affiliations:** 1 Department of Cardiology, John Radcliffe Hospital, Headington, Oxon, OX3 9DU, UK; 2 Department of Cardiology, Austin Health, Heidelberg, Victoria, 3084, Australia

## Abstract

**Background:**

Platypnea-orthodeoxia describes the condition of combined dyspnea and hypoxia respectively, whilst in the upright position, which improves in the recumbent position.

**Case Report:**

We present a case of platypnea-orthodeoxia due to a fenestrated atrial septal defect associated with an atrial septal aneurysm. Due to the fenestrated nature of the atrial septal defect, surgical rather than percutaneous correction was performed.

**Conclusion:**

A high index of suspicion is required to diagnose the syndrome of platypnea-orthodeoxia. Careful echocardiographic evaluation is required to identify the syndrome, and to determine suitability for percutaneous repair.

## Background

Persistence of a patent foramen ovale, or existence of an atrial septal defect, are both common and usually asymptomatic in the absence of right-to-left shunting, which usually occurs in the presence of elevated pulmonary artery and right heart pressures. Platypnea-orthodeoxia describes the condition of combined dyspnea and hypoxia respectively, whilst in the upright position, which improves in the recumbent position. The syndrome can occur with pulmonary or intra-cardiac shunts, however why right-to-left intra-cardiac shunting should only occur in the upright position is not fully understood.

### Case Report

A 75-year-old female presented with dyspnea. Her past medical history included hypertension and paroxysmal atrial fibrillation. Other than tachypnea, the cardiorespiratory examination was normal. The 12 lead electrocardiogram showed sinus rhythm with left atrial enlargement and non-specific T wave changes laterally. Arterial blood whilst breathing 12 litres of oxygen per minute via face mask was taken: pH 7.44, PaCO_2 _36 mmHg, PaO_2 _52 mmHg, O_2 _saturation 88%.

**Figure 1:**
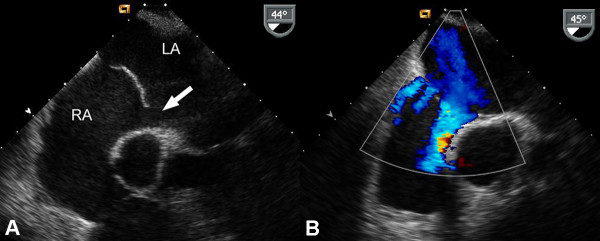
**Aneurysmal atrial septal defect. **(A) Transesophageal echocardiogram showing the left atrium (LA) and right atrium (RA). The atrial septal aneurysm can be seen bulging into the left atrium, and the ASD is shown (arrow). (B) Color flow Doppler across the atrial septum demonstrates multiple fenestrations with three distinct jets seen crossing the septum.

Full blood examination and D-Dimer were normal. A computed tomography pulmonary angiogram was normal. During admission there was fluctuating hypoxia, dyspnea and central cyanosis. An intra-cardiac shunt was suspected. A transesophageal echocardiogram (TEE) showed concentric left ventricular hypertrophy and normal right ventricular function. There was an atrial septal defect (ASD) with an aneurysmal interatrial septum (figure 1a), and mild left-to-right shunting only. Color flow Doppler across the atrial septum demonstrated multiple fenestrations (figure 1b). No right-to-left shunting was demonstrable with a saline contrast study, and a Swan Ganz catheter confirmed normal pulmonary artery pressures.

**Figure 2:**
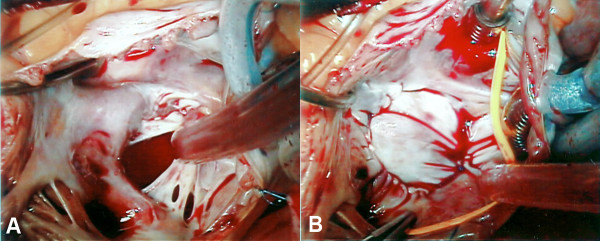
**Introperative findings. **(A) Intra-operative photograph demonstrating the fenestrated nature of the atrial septal defect, and the final result following surgical repair (B).

It was noted that her hypoxia was relieved in the recumbent position. On breathing room air whilst supine, the SpO_2 _was consistently above 90%, however in the sitting position, the SpO_2 _repeatedly dropped to as low as 70% with worsening dyspnea and development of central cyanosis. A repeat TEE was performed in both the supine and sitting position. At a 70 degree incline, there was significant right-to-left shunting. The aortic root appeared mildly dilated with distortion of the interatrial septum contributing to the shunt. Due to the fenestrated nature of the defect, surgical correction was performed (figures 2a and 2b). Twelve months later the patient is doing well without recurrence of symptoms.

## Conclusion

Platypnea-orthodeoxia has been described to occur in pulmonary arteriovenous shunts, pulmonary parenchymal shunts (as in the hepatopulmonary syndrome), or with intra-cardiac right-to-left shunts [1]. One of the earliest reported series of seven patients was published in 1984 [2]. Of these, four had prior pneumonectomy, and two had pulmonary embolism, although it was noted in each case that pulmonary hypertension was absent, and right-sided haemodynamics were normal. Since that time there have been a number of reports describing platypnea-orthodeoxia without overt lung disease. A review of 31 such cases from 1949–1997 was performed by Faller et al in 2000, and included platypnea-orthodeoxia associated with diaphragmatic paralysis, right atrial myxoma, restrictive cardiac disease, kyphoscoliosis and elongation of the ascending aorta [3]. Rare reports of platypnea-orthodeoxia in association with atrial septal aneurysms have mostly been associated with patent foramen ovale [3-5]. In our case, the syndrome was associated with an atrial septal aneurysm and ASD with multiple fenestrations – a complicating feature that precluded the patient from percutaneous closure, and which was confirmed at the time of surgical correction. Although the majority of defects are amenable to percutaneous intervention [6,7], careful evaluation of the atrial septum including the use of color flow Doppler is required to ensure suitability for percutaneous repair.

Several mechanisms have been theorized to cause right-to-left shunting in patients with atrial communications and normal right heart pressures. Theories include compression of the right atrium in the upright posture, decreased compliance of the right ventricle, and development of abnormal anatomy between the vena cava and the atrial septum [3,8]. The latter most likely contributes to the syndrome in our patient, with the mildly dilated aorta and atrial septal aneurysm permitting right-to-left shunting in the face of normal right-sided pressures.

Our case highlights two important aspects in the management of patients with orthostatic hypoxia and dyspnea. First there is the difficulty in making the diagnosis of right-to-left shunting in the presence of normal right heart and pulmonary artery pressures. Many patients will undergo investigation for pulmonary embolus and other alternative diagnoses. A high index of suspicion is therefore required to make a prompt diagnosis. The second is the need for careful echocardiographic evaluation with the use of controlled tilt to identify the syndrome, and determine the suitability for percutaneous repair.

Physicians should be aware of the syndrome of platypnea-orthodeoxia which is now a well recognized syndrome, with nearly 50 reported cases in the literature. A high index of suspicion is required to make the diagnosis, with echocardiography using controlled tilting the most useful investigational method. Careful evaluation of patients with respect to suitability for percutaneous repair is needed, as patients with fenestrated defects or large aneurysmal components may be best served with surgical correction to achieve complete closure of the septum, and ensure no persistence of right-to-left shunting.

## Competing interests

The author(s) declare that they have no competing interests.

## Authors' contributions

WVG: Conceived of the study, participated in the study design and drafted the manuscript. EJ, MJ: Performed the echocardiography, provided echocardiography images, participated in the study design and helped drafting the manuscript. GM, MH: Performed invasive procedures, provided surgical images and participated in the study design.
